# Invisible barriers: anthropogenic impacts on inter- and intra-specific interactions as drivers of landscape-independent fragmentation

**DOI:** 10.1098/rstb.2018.0049

**Published:** 2019-07-29

**Authors:** Oded Berger-Tal, David Saltz

**Affiliations:** Mitrani Department of Desert Ecology, Ben-Gurion University of the Negev, 8499000 Midreshet Ben Gurion, Israel

**Keywords:** behavioural syndromes, conservation behaviour, isolation by environment, landscape of fear, movement ecology, seed dispersal

## Abstract

Anthropogenically induced fragmentation constitutes a major threat to biodiversity. Presently, conservation research and actions focus predominantly on fragmentation caused directly by physical transformation of the landscape (e.g. deforestation, agriculture, urbanization, roads, etc.). While there is no doubt that landscape features play a key role in fragmenting populations or enhancing connectivity, fragmentation may also come about by processes other than the transformation of the landscape and which may not be readily visible. Such landscape-independent fragmentation (LIF) usually comes about when anthropogenic disturbance alters the inter- and intra-specific interactions among and within species. LIF and its drivers have received little attention in the scientific literature and in the management of wildlife populations. We discuss three major classes of LIF processes and their relevance for the conservation and management of species and habitats: (i) interspecific dispersal dependency, in which populations of species that rely on other species for transport and propagation become fragmented as the transporting species declines; (ii) interspecific avoidance induction, where species are excluded from habitats and corridors owing to interspecific interactions resulting from anthropogenically induced changes in community structure (e.g. exclusions by increased predation pressure); and (iii) intraspecific behavioural divergence, where populations become segregated owing to anthropogenically induced behavioural differentiation among them.

This article is part of the theme issue ‘Linking behaviour to dynamics of populations and communities: application of novel approaches in behavioural ecology to conservation'.

## 1. Background

One of the primary threats to biodiversity in the Anthropocene is habitat fragmentation [1,2]. Habitat fragmentation occurs when a continuous habitat is divided into two or more fragments associated with a consequent reduction in the total amount of area, as well as with changes to the habitat's spatial configuration [2–4]. Both processes may have devastating effects on animal populations by limiting dispersal (e.g. [5]), limiting access to food and mates (e.g. [6,7]), promoting Allee effects (e.g. [8,9]) and enhancing drift-related processes (e.g. [10,11]). Consequently, there is a huge body of research on habitat fragmentation, with some estimating that over 15% of ecological papers between the years 2000–2016 have dealt with habitat fragmentation or its inverse—habitat connectivity [12].

Habitat connectivity has been studied from two different perspectives [13]. The first, structural connectivity, refers to the physical configuration of the landscape (such as the shape, size and location of landscape features including physical barriers to movement; [14]). The second, functional connectivity, refers to how the behaviour of a dispersing individual is affected by landscape structure and elements, as expressed by its movement patterns [13]. Historic work on landscape connectivity tended to focus on structural connectivity. However, structural connectivity metrics are often meaningless for conservation planning when considered independently [13,15], and thus modern conservation work either centres on functional connectivity, or on the interactions between structural aspects of a landscape (such as vegetation type) and functional aspects of movement behaviour [16,17]. Regardless of the differences between the two approaches, both structural and functional connectivity are regarded as landscape metrics in their essence, derived directly from physical features of the area, with functional connectivity assumed to describe the ease at which animals can traverse some landscape type or feature regardless of the internal state of the dispersing individual or its motivation [17–19].

The field of population genetics has seen an even stronger shift away from landscape-centred metrics. Traditionally, the theory of 'isolation by distance' (IBD) was used to describe patterns in which genetic differentiation increases with geographical distance when dispersal among populations is geographically restricted [20]. Indeed, there is a large body of accumulated evidence showing IBD to be a common pattern in nature [21,22]. However, geography represents only one component that can potentially influence gene flow and population connectivity [23–25], and the past decade has seen the rise of the 'isolation by environment' (IBE) concept that describes a pattern in which genetic differentiation increases with environmental differences, independent of geographical distances [22,25,26]. However, IBE research tends to concentrate on describing patterns without investigating the mechanisms that have generated these patterns [25].

**Figure 1.**
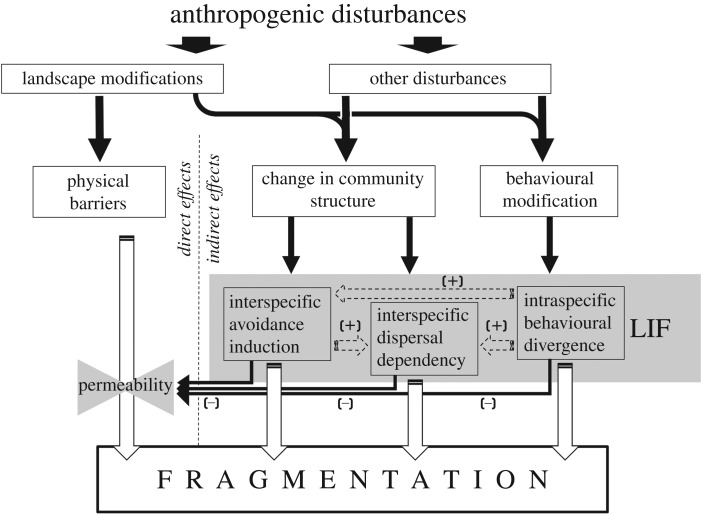
Population fragmentation can come about either owing to direct anthropogenic modifications to the landscape, or indirectly owing to the responses of animals to anthropogenically driven alteration to the community structure or owing to anthropogenic selection on animal behaviour. We term these indirect drivers landscape-independent fragmentation (LIF). Different LIF processes can interact and intensify other LIF processes, as well as interact and reduce the permeability of the physical landscape to some species.

While there is no doubt that landscape features play a key role in fragmenting populations or enhancing connectivity, fragmentation may also come about owing to processes not directly linked to the landscape. Such landscape-independent fragmentation (LIF) usually comes about when anthropogenic disturbance alters the inter- and intra-specific interactions among and within species. These altered interactions may change the way some species perceive their environment or use it, creating species-specific barriers that may be invisible to the human eye. Such processes have so far received little attention in the scientific literature and in the management of wildlife populations. In this paper, we will highlight three major classes of anthropogenically induced LIF processes: fragmentation resulting from interspecific dispersal dependency in which the decline of species that serve as pollinators or seed dispersers hinders the dispersal of the species that rely on them; fragmentation by interspecific exclusion where species avoid habitats and corridors owing to interspecific interactions such as increased predation risk or competition; and fragmentation by intraspecific behavioural segregation where populations become segregated owing to the effects of human-induced selection on behavioural traits (figure 1).

## 2. Interspecific dispersal dependency

Many species rely on other species for their population growth and propagation in space. Most notably, the majority of flowering plant species rely on animals for the transfer of male gametes to a receptive female plant, making pollination one of the most critical ecosystem processes on our planet [27,28]). Furthermore, many plants also rely on animals to disperse their seeds. This process, termed zoochory, is extremely common and can take many forms [29,30] such as transferring the seed in the gut of the animal (endozoochory; [31]), having the seed externally attached to a moving animal (epizoochory; [29,32]), or having the seed intentionally carried away by an animal for later consumption (synzoochory; e.g. [33]). Zoochory is a key process in forming, structuring and maintaining plant communities [34].

Plants that rely on pollination or seed dispersal for their propagation may, therefore, be affected by two types of fragmentation processes. The first, and already well-established process is landscape fragmentation that poses a real threat to pollination and seed dispersal systems by reducing the amount of resources available for pollinators and seed dispersers and by altering their community composition [27,28,35,36]. For example, Lehouck *et al.* [37] found that seed removal rates from intact forest patches were 3.5 higher on average than removal rates in fragmented patches, and fragmentation has been found to alter the fine-scale spatial genetic structure even for species that are wind-pollinated, owing to the prevention of secondary seed dispersal by rodents [38]. Second, the crucial role that pollinators and seed dispersers play in connecting between plants' populations means that any substantial decrease in their numbers, regardless of its cause, can greatly reduce connectivity among plant populations. This fragmentation process, which may be independent of any landscape features (e.g. it may result from overexploitation or disease), is expected to be especially pronounced in plants that specialize on a specific pollinator or seed disperser [35,39].

Wild bees, for example, are essential pollinators of many wild plants [28,40]. The global decline in bee populations worldwide [41,42] is bound to directly affect plant populations, as indeed some studies have already shown [41,43]. Other examples include the local extinction of Arabian oryx, *Oryx leucoryx*, in the beginning of the twentieth century. The oryx is the main disperser of *Acacia raddiana* seeds—a keystone species in the Negev Desert in Israel that provides habitat and food to many species, especially during the dry season. The disappearance of the oryx from the region has been suggested to contribute to recent die-offs of acacia stands, by extremely reducing the recruitment rate of new trees [44]. Similarly, in Kibale National Park, Uganda, seedling recruitment in forest patches where primates are harvested or driven away were dramatically lower than in patches with an intact frugivore community [45]. In some cases, changes to the behaviour of the seed disperser may be enough to drive fragmentation-inducing processes. For example, the Malabar grey hornbill, *Ocyceros griseus*, is a major seed disperser of white cedar, *Dysoxylum malabaricum*. However, despite being able to carry the *malabaricum*'s seed to distances of 10 km and more, in fragmented landscapes in the Western Ghats, 95% of seed dispersal was within 200 m of the fruiting tree, probably owing to changes in the movement patterns of the hornbills [46].

Fragmentation as a result of interspecific dispersal dependency has the same negative impacts as ‘regular’ landscape fragmentation such as reduced genetic diversity, increased sensitivity to Allee effects, and eventually extinction. However, in order to prevent interspecific dispersal dependency fragmentation from occurring, we must strive to conserve plant-pollinator and plant-seed disperser interactions whenever possible, and put an emphasis on specialist species, as these species are particularly vulnerable to environmental changes. In addition, in some cases locally extinct species can be reintroduced back into the ecosystem to restore their historic ecosystem functions as pollinators and seed-dispersers for local plant species [36,47].

## 3. Interspecific avoidance induction

Animal behaviour serves as a mediator between an individual and its environment. Consequently, changes to the environment are in many cases followed by behavioural changes aimed at mitigating any ill-effects the environmental changes may hold [48]. Fragmentation may occur when anthropogenic activity results in changes to the community structure (either directly or through cascading effects), and specifically, in an increased presence of predators or competitors owing to local overabundance, invasive aliens, or shifts in diel activity patterns [49].

In addition to the physical landscape which includes features such as mountains and valleys, running rivers and forests, there is another landscape, mostly invisible to the human eye; this is the ‘landscape of fear’ where the contours of the map represent different levels of perceived risk owing to interspecific aggressive interactions (mostly predation [50]). Predation risk is of course closely related to topographical features of the landscape, but is also strongly affected by many other factors such as the type of predator, the predator's mode of attack and overall lethality, the effectiveness of the prey's antipredator behaviours, etc. [50–53]. The landscape of fear shapes animals' habitat selection (e.g. European hares, *Lepus europaeus*, avoid riskier habitats with low vegetation for up to 24 h following predator exposure [54]) and movements across the 'real' landscape. Fear may affect dispersal patterns [55,56] either by promoting it (when the risk is high in the point of origin, e.g. [57,58]), or by hindering it (e.g. [59,60]), and may also shape the movement routes of migrating animals [61,62].

Anthropogenic disturbances can induce changes to animals' landscapes of fear, which in turn can influence their movement patterns and cause populations to become fragmented. For instance, human development is usually accompanied by substantial changes to wildlife communities, and in particular, with large shifts in the predators' community composition [63]. While many apex predators have been forced away from human-dominated landscapes (either to wilder areas, or to extinction), other predators thrive around humans. For example, coyotes, *Canis latrans*, are mesocarnivores that have been released from predation and competition with the eradication of large carnivores in the midwestern USA. As a result, their populations have increased dramatically [64,65], with serious consequences to their prey species. Jones *et al*. [65] demonstrated that coyotes' occurrences have instigated changes to the distribution and habitat use of various mammalian herbivores, causing white-tailed deer, *Odocoileus virginianus*, to seek denser forest cover, and squirrels (*Sciurus* spp.) and cottontails (*Sylvilagus floridanus*) to increase their use of urban areas. In Spain, the introduction of predators to the Ebro Delta region resulted in differential breeding dispersal in local Audouin's gull (*Ichthyaetus audouinii)* colonies: experienced breeders became more likely to leave the colony and younger breeders were more likely to stay. This differential dispersal modified the age structure in the colonies and reduced the reproductive success of the entire population [66].

Fragmentation may also come about through temporal changes to the landscape of fear. Human activity has a strong influence on animals' diel patterns, and animals across the globe have been documented to shift their activity period into the night in response to human disturbances [67]. Although diel shifts are in many cases central to maintain connectivity in species that avoid humans, the changes in the temporal patterns of one species owing to anthropogenic disturbance may cascade and impact the space-use patterns of other species, fragmenting their population in the process. For example, Shamoon *et al*. [49] used an array of camera-traps to measure the spatial and temporal activity patterns of five mammal species along a gradient of human activity in a Mediterranean natural-agricultural landscape, where the agricultural landscape is considered an ecological corridor by the planning authorities. They found out that human activity during the day in the agricultural fields caused a shift in the activity of the golden jackal, *Canis aureus*, to the night. The endangered mountain gazelle, *Gazella gazella*, also shifted its activity patterns in the presence of human disturbance, becoming much more active at night. However, in the agricultural fields, the presence of human activity during the day, and the resulting increased levels of predator activity during the night have practically excluded the gazelles from these areas, rendering them useless as ecological corridors for this species [49,68].

Humans do not only modify animal-movement behaviour by introducing predators into the landscape. In many cases, humans themselves are regarded as the predators. While this is of course evident in areas that suffer from high levels of hunting or poaching (e.g. [69]), some recreational activities also modify animals' landscapes of fear and constrain their movements [70,71].

So far, this section has focused predominantly on the effects of predation risk on animals' movement patterns. This is mainly owing to the fact that most known examples of interspecific interactions that limit and shape movement are centred on predator–prey interactions. However, other processes of interspecific avoidance inductions exist. One of these processes is interference competition. We know that many species alter their movement and habitat choice behaviours in order to avoid superior competitors. For instance, the presence of wolves limits the distribution and abundance of coyotes by altering the coyotes' dispersal rates [72], cheetahs actively move away from areas inhabited by lions and hyenas [73], and arctic foxes avoid areas with red foxes, thus being excluded from breeding in low altitude habitats [74]. Thus, interference competition can lead to population fragmentation through the same mechanism as predation risk. Another interspecific process that has been getting increased scientific attention recently is parasite avoidance behaviour that may be similar in many respects to anti-predatory behaviour. According to recent studies, the 'landscape of disgust', which represents animals' perceived infection threats, may hinder and shape the movement of individuals, the same way the landscape of fear does [75,76].

## 4. Intraspecific behavioural divergence

Anthropogenic disturbances exert human-induced selection [77,78]. Such anthropogenic selection can induce fragmentation by reducing effective dispersal as a result of behavioural barriers between populations. Because the type and intensity of anthropogenic disturbance vary over space and because this variation may be abrupt (e.g. a focal point highly visited by tourists within a larger natural and relatively pristine area), adjacent populations may be subject to different selection pressures. The role of animal behaviour as a mediator between the individual and its environment means that responses to these different selective forces will usually first be expressed via changes in individual behaviour which may, in turn, result in behavioural divergence and inter-population breeding avoidance, eventually generating genetic neighbourhoods [79]. Commensalism in urban environments, in particular, may drive such processes where the urban populations of synanthropic species may undergo local evolution favouring genotypes that are better fit to urban environments (e.g. noise adaptations) and those exhibiting increased dependence on anthropogenic resources [80,81], while their conspecific outside the urban areas remain relatively unchanged.

Bird singing dialects are a good example of how such processes may be manifested: Danner *et al*. [82] found that females of rufous-collared sparrows (*Zonotrichia capensis*) prefer to mate with males singing natal dialects thereby driving reproductive isolation. At the same time, several studies reported that urban birds change their dialect to cope with the noisy environments [83] and males responded more strongly to current than to historical songs [84]. Such behavioural divergence between bird populations can lead to genetic differentiation despite the fact that no physical barrier separates them from one other. Another phenomenon that may induce behavioural divergence and generate LIF is habituation and the resulting dependence of wildlife populations on human resources. In Israel, commensal foxes have access to abundant resources and consequently are characterized by high densities, smaller home-range size, heavier offspring, and higher recruitment rates [85]. Although individuals in these populations emigrate out into the natural surroundings, they are unable to survive in the wild [86,87] and with little evidence of any immigration, they appear to be isolated from the fox populations in the surrounding matrix.

A similar example comes from Switzerland, where allelic diversity and genetic differentiation were quantified between urban and rural populations of red foxes, *Vulpes vulpes*, around Zurich [88]. The researchers found that the urban fox populations, which at the time of the test were approximately 15 years old, were differentiated genetically from nearby rural populations. The observed differentiation could not be explained by the geographical arrangement of the populations because genetic differentiation between two rural populations was much lower despite the fact that the geographical distance was much higher between them. Genetic differentiation among close populations has been documented in other species as well. For instance, the Nubian ibex, *Capra nubiana*, is a social desert ungulate that is listed as Vulnerable by the International Union for Conservation of Nature, and is typically found on or nearby cliffs in the vicinity of desert oases. Ibex populations in the vicinity of two human settlements in the Negev desert in Israel (the village of Midreshet Ben-Gurion and the town of Mitzpe Ramon) have been shown to exhibit high tolerance towards humans and human disturbances [89], and an increasing reliance on food and water resources provided by these settlements (D. Saltz 2019, personal observations). The distance between the two settlements is approximately 50 km that include continuous cliffs and water sources, both of which are important for the ibex, and in the past, the presence of ibex has been regularly documented on this route [90]. However, a recent analysis found strong genetic differentiation between the two populations [90]. Because the physical landscape in the area did not undergo any major alterations in the past few decades, the separation between the two populations is believed to be caused by behavioural differentiation because of their attraction to the human settlements [90]. Both of the abovementioned examples (the foxes and the ibex), are cases of documented 'isolation by environment', where genetic differentiation is a function of the environment and is independent of geographical distance. The proposed mechanism in these cases is behavioural segregation or behavioural specialization where a positive feedback loop is created—becoming specialized in one habitat reduces the tendency to use other habitat types, which further increases specialization and so forth.

Dispersal is a complex behaviour that is composed from a suite of phenotypic traits (also called dispersal syndromes; [91]), and is correlated with many other internal and external factors, including other behavioural traits. Similarly, a behavioural syndrome is a suite of correlated behaviours expressed across different contexts (e.g. correlations among feeding, antipredator, mating, aggressive and dispersal behaviours; [92]). Recently, there is a growing awareness in the scientific literature of the links between dispersal syndromes and behavioural syndromes (also called personality-dependent dispersal syndromes; [93]). For example, boldness is usually associated with increased dispersal [94,95]. The effects of aggression on dispersal behaviour are less clear-cut [95,96]. In some species, less aggressive individuals were found to be more likely to disperse [97–99], while in other species, more aggressive individuals were more dispersive [100,101]. Other more specific behaviours have also been associated with dispersal behaviours. For instance, in the Alpine swift, *Apus melba*, nest-defence behaviour has been found to be negatively correlated with natal dispersal [102]. This means that anthropogenic selection against some behavioural traits (such as boldness) can result in increased fragmentation, because shyer individuals are less likely to disperse. Moreover, such selection may result in synergistic interactions with other fragmentation-inducing processes. For instance, shyer individuals may also be more likely to avoid corridors owing to interspecific avoidance induction.

## 5. Discussion

We highlighted three landscape-independent processes that can fragment wild populations of animals and plants (table 1). It is important to point out that we have separated the processes governing fragmentation into landscape and landscape-independent in order to draw attention to important processes that have so far received very little attention in the literature. However, in reality, it is likely that the separation into landscape and landscape-independent processes is murkier. For example, wolves are known to prefer to travel along natural and anthropogenic linear features causing woodland caribou in northeastern Alberta to avoid such features [103]. The caribou are reacting to a change in the community structure in their area, avoiding areas of higher predation risk, but this change is directly linked to anthropogenic modifications of the landscape. In a similar fashion, animals may be deterred by traffic noise or the road lights, rather than by the road itself, because both noise and light can be associated with heightened predation risk [104,105]. Another case that serves to emphasize the complex interactions between landscape dependent and independent processes is the effects of local resources on interspecific dispersal dependency. Any changes to the distribution of attractive resources—such as food patches, shelters or conspecifics, or alternatively, any changes to the way individuals *perceive* these resources, may alter movement of individuals through the transformed landscape, which in turn may have a strong effect on plant species relying on these individuals for spatial propagation. We, therefore, believe that anthropogenic fragmentation may be best described as a continuum in which at one end fragmentation is purely the result of landscape modifications that present physical barriers to movement, and at the other end, fragmentation is purely the result of anthropogenic impacts on inter- and intra-specific interactions without any landscape component to them (figure 1).

**Table 1.** A list of landscape-independent fragmentation (LIF) processes that are highlighted in this paper, their main drivers, and the main mechanisms through which they can elicit fragmentation.

LIF process	driver	main mechanisms
interspecific dispersal dependency	changes to community structure	—global or local extinction of a pollinator, seed disperser or host species —reduced movement in a pollinator, seed disperser or host species
interspecific avoidance induction	changes to community structure	—‘landscape of fear': avoidance of areas (and periods) with high perceived risk of predation —avoidance of areas (and periods) with strong interference competition —‘landscape of disgust’: avoidance of areas (and periods) with high perceived infection rates
intraspecific behavioural divergence	anthropogenic selection on behaviour	—selection for reduced dispersal —selection for behaviours linked to reduced dispersal (e.g. shyness, low aggressiveness) —divergence in courtship or mating behaviour —divergence owing to high tolerance of local populations to human disturbances

It is also important to note that LIF processes are not mutually exclusive and they may operate at the same time or may be dependent on one another resulting either in synergism (see example above on how selection against boldness can induce fragmentation through two different processes), or in a cascading fragmentation effect (e.g. if dispersal is reduced in species A owing to interspecific avoidance induction, this can also induce fragmentation in species B, if species B relies on species A for propagation or transport).

In this paper, we concentrate on how inter- and intra-specific interactions can drive LIF, but the same general principles we put forth can also explain landscape-independent increase in connectivity. From the perspective of interspecific dispersal dependency, novel and exotic species can serve as new seed dispersers or pollinators [106,107], and humans themselves can be a major agent of transportation for pollen, seeds and entire organisms [108]. Interspecific avoidance induction may sometimes become interspecific attraction induction when anthropogenic changes to the community increase the propensity of species to move into certain habitats, and just as human-induced selection could reduce the tendency of animals to disperse, it may also make that tendency stronger in some cases [109]. From a conservation perspective, such 'invisible movement facilitators' may benefit populations at risk, but may also pose a serious problem when they promote the movement of invasive species and disease vectors [110,111]. As in the case of the invisible barriers, being aware of the processes that promote movement across the landscape is the first step in creating effective mitigation strategies when necessary.

The movement and habitat choices of animals are directly linked to the distribution of animal and plant species in space and time [48,112]. Therefore, any changes to these movement patterns are bound to have an impact on their ecosystem. These 'behavioural cascades' have mostly been described in the literature in the context of the non-lethal effects of top predators on their ecosystems (e.g. [48,49,113]) and are also known as behaviourally mediated trophic cascades (BMTCs, [114,115]). Indeed, the process of interspecific avoidance induction encapsulates some of the negative consequences that human-induced BMTCs can have on wildlife populations. However, behavioural cascades are not limited to the effects of apex predators. Novel anthropogenic resources, such as dumps, crop residuals, and fish ponds are relatively predictable in space and time, making them sometimes easier to access than natural resources [116]. Such resources have been found to alter the movement and space-use behaviours of predators and prey alike [117,118], thus initiating behavioural cascades that can radically change ecosystems. Other anthropogenic disturbances such as noise or light pollution can also elicit behavioural changes that can cascade throughout ecosystems [119,120]. We believe that studies investigating behavioural cascades that are driven by anthropogenic changes to the landscape are sorely needed.

There is no doubt that understanding how animal movement interacts with the physical attributes of the landscape is critical for our ability to conserve and manage wildlife populations. However, it may not be enough. To successfully and efficiently address the problem of fragmentation, we must integrate landscape-independent genetic and behavioural knowledge with the more traditional landscape-based approach. By gaining insights into the mechanistic underpinnings of movement and dispersal, wildlife managers will be able to identify and eliminate or mitigate LIF processes, increasing connectivity among populations, thus improving their chances of survival in our rapidly changing world.

## Data accessibility

This article has no additional data.

## References

[1] Groom MJ, Meffe GK, Carroll CR 2006 Principles of conservation biology, 4th edn Sunderland, MA: Sinauer Associates.

[2] Primack RB 2014 Essentials of conservation biology, 6th edn Sunderland, MA: Sinauer Associates.

[3] Fahrig L 2003 Effects of habitat fragmentation on biodiversity. Annu. Rev. Ecol. Evol. Syst. 34, 487–515. (10.1146/annurev.ecolsys.34.011802.132419)

[4] Noss RF 1991 Landscape connectivity: different functions at different scales. In Landscape linkages and biodiversity (ed. Hudson WE), pp. 27–39. Washington, DC: Island Press.

[5] Stouffer PC, Johnson EI, Bierregaard RO Jr, Lovejoy TE 2011 Understory bird communities in Amazonian rainforest fragments: species turnover through 25 years post-isolation in recovering landscapes. PLoS ONE 6, e20543 (10.1371/journal.pone.0020543) 21731616 PMC3120763

[6] Banks SC, Piggott MP, Stow AJ, Taylor AC 2007 Sex and sociality in a disconnected world: a review of the impacts of habitat fragmentation on animal social interactions. Can. J. Zool. 85, 1065–1079. (10.1139/Z07-094)

[7] Becker CG, Fonseca CR, Haddad CFB, Prado PI 2010 Habitat split as a cause of local population declines of amphibians with aquatic larvae. Con. Biol. 24, 287–294. (10.1111/j.1523-1739.2009.01324.x) 19758391

[8] Lamont BB, Klinkhamer PGL, Witkowski ETF 1993 Population fragmentation may reduce fertility to zero in *Banksia goodie* – a demonstration of the Allee effect. Oecologia 94, 446–450. (10.1007/BF00317122) 28313684

[9] Hanski I, Ovaskainen O 2003 Metapopulation theory for fragmented landscapes. Theor. Popul. Biol. 64, 119–127. (10.1016/S0040-5809(03)00022-4) 12804876

[10] Keller I, Largiader CR 2003 Recent habitat fragmentation caused by major roads leads to reduction of gene flow and loss of genetic variability in ground beetles. Proc. R. Soc. Lond. B 270, 417–423. (10.1098/rspb.2002.2247) PMC1691256 12639322

[11] Vranckx G, Jacquemyn H, Muys B, Honnay O 2012 Meta-analysis of susceptibility of woody plants to loss of genetic diversity through habitat fragmentation. Con. Biol. 26, 228–237. (10.1111/j.1523-1739.2011.01778.x) 22044646

[12] Fardila D, Kelly LT, Moore JL, McCarthy MA 2017 A systematic review reveals changes in where and how we have studied habitat loss and fragmentation over 20 years. Biol. Con. 212, 130–138. (10.1016/j.biocon.2017.04.031)

[13] Baguette M, Van Dyck H 2007 Landscape connectivity and animal behavior: functional grain as a key determinant for dispersal. Landscape Ecol. 22, 1117–1129. (10.1007/s10980-007-9108-4)

[14] Brooks CP 2003 A scalar analysis of landscape connectivity. Oikos 102, 433–439. (10.1034/j.1600-0579.2003.11511.x)

[15] Calabrese JM, Fagan WF 2004 A comparison shoppers guide to connectivity metrics: trading off between data requirements and information content. Front. Ecol. Environ. 2, 529–536. (10.1890/1540-9295(2004)002[0529:ACGTCM]2.0.CO;2)

[16] Taylor PD, Fahrig L, Henein K, Merriam G 1993 Connectivity is a vital element in landscape structure. Oikos 68, 571–573. (10.2307/3544927)

[17] Clair CCS, Found R, Gangadharan A, Murray M 2016 Behavior-based contributions to reserve design and management. In Conservation behavior: applying behavioral ecology to wildlife conservation and management (eds Berger-Tal O, Saltz D), pp. 176–211. Cambridge, UK: Cambridge University Press.

[18] Nathan R, Getz WM, Revilla E, Holyoak M, Kadmon R, Saltz D, Smouse PE 2008 A movement ecology paradigm for unifying organismal movement research. Proc. Natl Acad. Sci. USA 105, 19 052–19 059. (10.1073/pnas.0800375105) PMC2614714 19060196

[19] Wittemyer G, Northrup JM, Bastille-Rousseau G 2019 Behavioural valuation of landscapes using movement data. Pil. Trans. R. Soc. B 374, 20180046 (10.1098/rstb.2018.0046) PMC6710572 31352884

[20] Slatkin M 1993 Isolation by distance in equilibrium and nonequilibrium populations. Evolution 47, 264–279. (10.1111/j.1558-5646.1993.tb01215.x) 28568097

[21] Meirmans PG 2012 The trouble with isolation by distance. Mol. Ecol. 21, 2839–2846. (10.1111/j.1365-294X.2012.05578.x) 22574758

[22] Sexton JP, Hangartner SB, Hoffmann AA 2013 Genetic isolation by environment or distance: which pattern of gene flow is most common? Evolution 68, 1–15. (10.1111/evo.12258) 24111567

[23] Crispo E, Bentzen P, Reznick DN, Kinnison MT, Hendry AP 2006 The relative influence of natural selection and geography on gene flow in guppies. Mol. Ecol. 15, 49–62. (10.1111/j.1365-294X.2005.02764.x) 16367829

[24] Lee C-R, Mitchell-Olds T 2011 Quantifying effects of environmental and geographical factors on patterns of genetic differentiation. Mol. Ecol. 20, 4631–4642. (10.1111/j.1365-294X.2011.05310.x) 21999331 PMC3204174

[25] Wang IJ, Bradburd GS 2014 Isolation by environment. Mol. Ecol. 23, 5649–5662. (10.1111/mec.12938) 25256562

[26] Wang IJ, Summers K 2010 Phenotypic divergence rather than geographic isolation in the highly polymorphic strawberry poison-dart frog. Mol. Ecol. 19, 447–458. (10.1111/j.1365-294X.2009.04465.x) 20025652

[27] Rathcke BJ, Jules ES 1993 Habitat fragmentation and plant-pollinator interactions. Curr. Sci. 65, 273–277.

[28] Kearns CA, Inouye DW, Waser NM 1998 Endangered mutualisms: the conservation of plant-pollinator interactions. Annu. Rev. Ecol. Syst. 29, 83–112. (10.1146/annurev.ecolsys.29.1.83)

[29] Howe HF, Smallwood J 1982 Ecology of seed dispersal. Annu. Rev. Ecol. Syst. 13, 201–228. (10.1146/annurev.es.13.110182.001221)

[30] Groom PK, Lamont BB 2015 Plant life of southwestern Australia. Adaptations for survival. Warsaw/Berlin, Poland/Germany: De Gruyter Open Ltd.

[31] Corlett RT 1998 Frugivory and seed dispersal by vertebrates in the Oriental (Indomalayan) region. Biol. Rev. 73, 413–448. (10.1017/S0006323198005234) 9951414

[32] Sorensen AE 1986 Seed dispersal by adhesion. Annu. Rev. Ecol. Syst. 17, 443–463. (10.1146/annurev.es.17.110186.002303)

[33] Lengyel S, Gove AD, Latimer AM, Majer JD, Dunn RR 2010 Convergent evolution of seed dispersal by ants, and phylogeny and biogeography in flowering plants: a global survey. Perspect. Plant. Ecol. Syst. 12, 43–55. (10.1016/j.ppees.2009.08.001)

[34] Corlett RT 2017 Frugivory and seed dispersal by vertebrates in tropical and subtropical Asia: an update*.* Glob. Ecol. Conserv. 11, 1–22. (10.1016/j.gecco.2017.04.007)

[35] Kwak MM, Velterop O, van Andel J 1998 Pollen and gene flow in fragmented habitats. Appl. Veg. Sci. 1, 37–54. (10.2307/1479084)

[36] McConkey KR, Prasad S, Corlett RT, Campos-Arceiz A, Brodie JF, Rogers H, Santamaria L 2012 Seed dispersal in changing landscapes. Biol. Con. 146, 1–13. (10.1016/j.biocon.2011.09.018)

[37] Lehouck V, Spanhove T, Colson L, Adringa-Davis A, Cordeiro NJ, Lens L 2009 Habitat disturbance reduces seed dispersal of a forest interior tree in a fragmented African cloud forest. Oikos 118, 1023–1034. (10.1111/j.1600-0706.2009.17300.x)

[38] Wang R, Compton SG, Chen X-Y 2011 Fragmentation can increase spatial genetic structure without decreasing pollen-mediated gene flow in a wind-pollinated tree. Mol. Ecol. 20, 4421–4432. (10.1111/j.1365-294X.2011.05293.x) 21981067

[39] Weiner CN, Werner M, Linsenmair KE, Bluthgen N 2014 Land-use impacts on plant-pollinator networks: interaction strength and specialization predict pollinator declines. Ecology 95, 466–474. (10.1890/13-0436.1) 24669739

[40] Corbet SA, Williams IH, Osborne JL 1991 Bees and the pollination of crops and wild flowers in the European community. Bee World 72, 47–59. (10.1080/0005772X.1991.11099079)

[41] Biesmeijer JC et al. 2006 Parallel declines in pollinators and insect-pollinated plants in Britain and the Netherlands. Science 313, 351–354. (10.1126/science.1127863) 16857940

[42] Everaars J, Settele J, Dormann CF 2018 Fragmentation of nest and foraging habitat affects time budgets of solitary bees, their fitness and pollination services, depending on traits: results from an individual-based model. PLoS ONE 13, e0188269 (10.1371/journal.pone.0188269) 29444076 PMC5812554

[43] Burkle LA, Marlin JC, Knight TM 2013 Plant-pollinator interactions over 120 years: loss of species, co-occurrence and function. Science 339, 1611–1615. (10.1126/science.1232728) 23449999

[44] Polak T, Gutterman Y, Hoffman I, Saltz D 2014 Redundancy in seed dispersal by three sympatric ungulates: a reintroduction perspective. Anim. Con. 17, 565–572. (10.1111/acv.12122)

[45] Chapman CA, Onderdonk DA 1998 Forests without primates: primate/plant codependency. Am. J. Primatol. 45, 127–141. (10.1002/(SICI)1098-2345(1998)45:1<127::AID-AJP9>3.0.CO;2-Y) 9573446

[46] Ismail SA, Ghazoul J, Ravikanth G, Kushalappa CG, Shaanker RU, Kettle CJ 2017 Evaluating realized seed dispersal across fragmented tropical landscapes: a two-fold approach using parentage analysis and the neighbourhood model. New Phytol. 214, 1307–1316. (10.1111/nph.14427) 28134981

[47] Polak T, Saltz D 2011 Reintroduction as an ecosystem restoration technique. Con. Biol. 25, 424–427. (10.1111/j.1523-1739.2011.01669.x) 21535145

[48] Berger-Tal O, Saltz D 2016 Conservation behavior: applying behavioral ecology to wildlife conservation and management. Cambridge, UK: Cambridge University Press.

[49] Shamoon H, Maor R, Saltz D, Dayan T 2018 Increased mammal nocturnality in agricultural landscapes results in fragmentation due to cascading effects. Biol. Con. 226, 32–41. (10.1016/j.biocon.2018.07.028)

[50] Laundré JW, Hernandez L, Altendorf KB 2001 Wolves, elk, and bison: re-establishing the “landscape of fear” in Yellowstone National Park, USA. Can. J. Zool. 79, 1401–1409. (10.1139/z01-094)

[51] Brown JS, Laundré JW, Gurung M 1999 The ecology of fear: optimal foraging, game theory, and trophic interactions. J. Mammal. 80, 385–399. (10.2307/1383287)

[52] Brown JS, Kotler BP 2004 Hazardous duty pay and the foraging cost of predation. Ecol. Lett. 7, 999–1014. (10.1111/j.1461-0248.2004.00661.x)

[53] Kotler BP, Morris DW, Brown JS 2016 Direct behavioral indicators as a conservation and management tool. In Conservation behavior: applying behavioral ecology to wildlife conservation and management (eds Berger-Tal O, Saltz D), pp. 307–351. Cambridge, UK: Cambridge University Press.

[54] Weterings MJA, Zaccaroni M, van der Koore N, Zijlstra LM, Kuipers HJ, van Langevelde F, van Wieren SE 2016 Strong reactive movement response of the medium-sized European hare to elevated predation risk in short vegetation. Anim. Behav. 115, 107–114. (10.1016/j.anbehav.2016.03.011)

[55] Bonnet X, Naulleau G, Shine R 1999 The dangers of leaving home: dispersal and mortality in snakes. Biol. Con. 89, 39–50. (10.1016/S0006-3207(98)00140-2)

[56] Bonte D et al. 2012 Costs of dispersal. Biol. Rev. 87, 290–312. (10.1111/j.1469-185X.2011.00201.x) 21929715

[57] Hakkarainen H, Ilmonen P, Koivunen V, Korpimaki E 2001 Experimental increase of predation risk induces breeding dispersal of Tengmalm's owl. Oecologia 126, 355–359. (10.1007/s004420000525) 28547448

[58] Cronin JT, Haynes KJ, Dillemuth F 2004 Spider effects on planthopper mortality, dispersal, and spatial population dynamics. Ecology 85, 2134–2143. (10.1890/03-0591)

[59] Heg D, Bachar Z, Brouwer L, Taborsky M 2004 Predation risk is an ecological constraint for helper dispersal in a cooperatively breeding cichlid. Proc. R. Soc. Lond. B 271, 2367–2374. (10.1098/rspb.2004.2855) PMC1691868 15556889

[60] Cortez MV, Navarro JL, Martella MB 2018 Effect of antipredator training on spatial behaviour of male and female greater rheas, *Rhea Americana*, reintroduced into the wild. Acta Ornithol. 53, 81–90. (10.3161/00016454AO2018.53.1.008)

[61] Avgar T et al. 2015 Space-use behaviour of woodland caribou based on a cognitive movement model. J. Anim. Ecol. 84, 1059–1070. (10.1111/1365-2656.12357) 25714592

[62] Paton DG, Ciuti S, Quinn M, Boyce MC 2017 Hunting exacerbates the response to human disturbance in large herbivores while migrating through a road network. Ecoshpere 8, 1–18. (10.1002/ecs2.1841)

[63] Ritchie EG, Johnson CN 2009 Predator interactions, mesopredator release and biodiversity conservation. Ecol. Lett. 12, 982–998. (10.1111/j.1461-0248.2009.01347.x) 19614756

[64] Prugh LR, Stoner CJ, Epps CW, Bean WT, Ripple WJ, Laliberte AS, Brashares JS 2009 The rise of the mesopredator. BioScience 59, 779–791. (10.1525/bio.2009.59.9.9)

[65] Jones BM, Cove MV, Lashley MA, Jackson VL 2016 Do coyotes, *Canis latrans*, influence occupancy of prey in suburban forest fragments? Curr. Zool. 62, 1–6. (10.1093/cz/zov004) 29491884 PMC5804128

[66] Payo-Payo A, Sanz-Aguilar A, Genovart M, Bertolero A, Piccardo J, Camps D, Ruiz-Olmo J, Oro D 2018 Predator arrival elicits differential dispersal, change in age structure and reproductive performance in a prey population. Sci. Rep. 8, 1971 (10.1038/s41598-018-20333-0) 29386550 PMC5792507

[67] Gaynor KM, Hojnowski CE, Carter NH, Brashares JS 2018 The influence of human disturbance on wildlife nocturnality. Science 360, 1232–1235. (10.1126/science.aar7121) 29903973

[68] Shamoon H, Saltz D, Dayan T 2017 Fine-scale temporal and spatial population fluctuations of medium sized carnivores in a Mediterranean agricultural matrix. Landscape Ecol. 32, 1243–1256. (10.1007/s10980-017-0517-8)

[69] Thurfjell H, Ciuti S, Boyce MS 2017 Learning from the mistakes of others: how female elk (*Cervus elaphus*) adjust behaviour with age to avoid hunters. PLoS ONE 12, e0178082 (10.1371/journal.pone.0178082) 28614406 PMC5470680

[70] Rosner S, Mussard-Forster E, Lorenc T, Muller J 2014 Recreation shapes a “landscape of fear“ for a threatened forest bird species in Central Europe. Landscape Ecol. 29, 55–66. (10.1007/s10980-013-9964-z)

[71] Richard JH, Cote SD 2016 Space use analyses suggest avoidance of a ski area by mountain goats. J. Wildl. Manage. 80, 387–395. (10.1002/jwmg.1028)

[72] Berger KM, Gese EM 2007 Does interference competition with wolves limit the distribution and abundance of coyotes? J. Anim. Ecol. 76, 1075–1085. (10.1111/j.1365-2656.2007.01287.x) 17922704

[73] Durant SM 2000 Living with the enemy: avoidance of hyenas and lions by cheetahs in the Serengeti. Behav. Ecol. 11, 625–632. (10.1093/beheco/11.6.624)

[74] Tannerfeldt M, Elmhagen B, Angerbjorn A 2002 Exclusion by interference competition? The relationship between red and arctic foxes. Oecologia 132, 213–220. (10.1007/s00442-002-0967-8) 28547354

[75] Buck JC, Weinstein SB, Young HS 2018 Ecological and evolutionary consequences of parasite avoidance. Trends Ecol. Evol. 33, 619–632. (10.1016/j.tree.2018.05.001) 29807838

[76] Weinstein SB, Buck JC, Young HS 2018 A landscape of disgust. Science 359, 1213–1214. (10.1126/science.aas8694) 29590062

[77] Allendorf FW, Hard JJ 2009 Human-induced evolution caused by unnatural selection through harvest of wild animals. Proc. Natl Acad. Sci. USA 106, 9987–9994. (doi:10.1073_pnas.0901069106) 19528656 10.1073/pnas.0901069106 PMC2702803

[78] Shefferson RP, Mason CM, Kellett KM, Goolsby EW, Coughlin E, Flynn RW 2018 The evolutionary impacts of conservation actions*.* Pop. Ecol. 60, 49–59. (10.1007/s10144-018-0614-9)

[79] Schaal BA 1980 Measurment of gene flow in *Lupinus texensis*. Nature 284, 450–451. (10.1038/284450a0)

[80] Lowry H, Lill A, Wong BBM 2013 Behavioural responses of wildlife to urban environments. Biol. Rev. 88, 537–549. (10.1111/brv.12012) 23279382

[81] Hulme-Beaman A, Dobney K, Cucchi T, Searle JB 2016 An ecological and evolutionary framework for commensalism in anthropogenic environments. Trends Ecol. Evol. 31, 633–645. (10.1016/j.tree.2016.05.001) 27297117

[82] Danner JE, Danner RM, Bonier F, Martin PR, Small TW, Moore IT 2011 Female, but not male, tropical sparrows respond more strongly to the local song dialect: implications for population divergence. Am. Nat. 178, 53–63. (10.1086/660283) 21670577

[83] Luther DA, Baptista L 2010 Urban noise and the cultural evolution of bird songs. Proc. R. Soc. B 277, 469–473. (10.1098/rspb.2009.1571) PMC2842653 19846451

[84] Luther DA, Derryberry EP 2012 Birdsongs keep pace with city life: changes in song over time in an urban songbird affects communication. Anim. Behav. 83, 1059–1066. (10.1016/j.anbehav.2012.01.034)

[85] Dolev A, Saltz D, King R 2006 Anthropogenic-dependent over-abundance of fox populations: patterns and conservation implications. In Page 23 in book of abstracts, 1st European Congress of Conservation Biology, Eger, Hungary See http://www.carnivoreconservation.org/files/meetings/eccb_2006_eger_full.pdf.

[86] Bino G, Dolev A, Yosha D, Guter A, King R, Saltz D, Kark S 2010 Abrupt spatial and numerical responses of overabundant foxes to a reduction in anthropogenic resources. J. App. Ecol. 47, 1262–1271. (10.1111/j.1365-2664.2010.01882.x)

[87] Kapota D, Dolev A, Bino G, Yosha D, Guter A, King R, Saltz D 2016 Determinants of emigration and their impact on survival during dispersal in fox and jackal populations*.* Sci. Rep. 6, 24021 (10.1038/srep24021) 27050564 PMC4822138

[88] Wandeler P, Funk SM, Largiader CR, Gloor S, Breitenmoser U 2003 The city-fox phenomenon: genetic consequences of a recent colonization of urban habitat. Mol. Ecol. 12, 647–656. (10.1046/j.1365-294X.2003.01768.x) 12675821

[89] Saltz D, Berger-Tal O, Motro U, Shkedy Y, Raanan N 2019 Conservation implications of habituation as determined by changes in the vigilance/group-size effect of Nubian ibex in response to ecotourism. Anim. Con. 22, 220–227. (10.1111/acv.12456)

[90] Goodman I 2016 The spatial genetic structure of the Nubian ibex (*Capra nubiana*) population in Israel. MSc Thesis, Ben-Gurion University of the Negev, Israel.

[91] Clobert J, Le Galliard J-F, Cote J, Meylan S, Massot M 2009 Informed dispersal, heterogeneity in animal dispersal syndromes and the dynamics of spatially structured populations. Ecol. Lett. 12, 197–209. (10.1111/j.1461-0248.2008.01267.x) 19170731

[92] Sih A, Bell AM, Johnson JC, Ziemba RE 2004 Behavioral syndromes: an integrative overview. Q. Rev. Biol. 79, 241–277. (10.1086/422893) 15529965

[93] Spiegel O, Leu ST, Bull CM, Sih A 2017 What's your move? Movement as a link between personality and spatial dynamics in animal populations. Ecol. Lett. 20, 3–18. (10.1111/ele.12708) 28000433

[94] Fraser DF, Gilliam JF, Daley MJ, Le AN, Skalski GT. 2001 Explaining leptokurtic movement distributions: intrapopulation variation in boldness and exploration. Am. Nat. 158, 124–135. (10.1086/321307) 18707341

[95] Cote J, Clobert J, Brodin T, Fogarty S, Sih A 2010 Personality-dependent dispersal: characterization, ontogeny and consequences for spatially structured populations. Phil. Trans. R. Soc. B 365, 4065–4076. (10.1098/rstb.2010.0176) 21078658 PMC2992741

[96] Cooper EB, Taylor RW, Kelley AD, Martining AR, Boutin S, Humphries MM, Dantzer B, Lane JE, McAdam AG 2017 Personality is correlated with natal dispersal in North American red squirrels (*Tamiasciurus hudsonicus*). Behaviour 154, 939–961. (10.1163/1568539X-00003450)

[97] Schradin C, Lampprecht J 2002 Causes of female emigration in the group-living cichlid fish *Neolamprologus multifasciatus**.* Ethology 108, 237–248. (10.1046/j.1439-0310.2002.00775.x)

[98] Pocock MJO, Hauffe HC, Searle JB 2005 Dispersal in house mice. Biol. J. Linnean Soc. 84, 565–583. (10.1111/j.1095-8312.2005.00455.x)

[99] Guerra PA, Pollack GS 2010 Colonists and desperadoes: different fighting strategies in wing-dimorphic male Texas field crickets. Anim. Behav. 79, 1087–1093. (10.1016/j.anbehav.2010.02.002)

[100] Duckworth RA, Badyaev AV 2007 Coupling of dispersal and aggression facilitates the rapid range expansion of a passerine bird. Proc. Natl Acad. Sci. USA 104, 15 017–15 022. (10.1073/pnas.0706174104) 17827278 PMC1986605

[101] Aguillon SM, Duckworth RA 2015 Kin aggression and resource availability influence phenotype-dependent dispersal in a passerine bird. Behav. Ecol. Sociobiol. 69, 625–633. (10.1007/s00265-015-1873-5)

[102] Bize P, Daniel G, Viblanc VA, Martin JGA, Doligez B 2017 Negative phenotypic and genetic correlation between natal dispersal propensity and nest-defence behaviour in a wild bird. Biol. Lett. 13, 2017236 (10.1098/rsbl.2017.0236) PMC5543023 28747532

[103] Latham ADM, Latham MC, Boyce MS, Boutin S 2011 Movement responses by wolves to industrial linear features and their effect on woodland caribou in northeastern Alberta. Ecol. App. 21, 2854–2865. (10.1890/11-0666.1)

[104] McClure CJW, Ware HE, Carlisle J, Kaltenecker G, Barber JR 2013 An experimental investigation into the effects of traffic noise on distribution of birds: avoiding the phantom road. Proc. R. Soc. B 280, 20132290 (10.1098/rspb.2013.2290) PMC3826227 24197411

[105] Bliss-Ketchum LL, de Rivera CE, Turner BC, Weisbaum DM 2016 The effect of artificial light on wildlife use of a passage structure. Biol. Con. 199, 25–28. (10.1016/j.biocon.2016.04.025)

[106] Foster JT, Robinson SK 2007 Introduced birds and the fate of Hawaiian rainforests. Con. Biol. 21, 1248–1257. (10.1111/j.1523-1739.2007.00781.x) 17883490

[107] Shiels AB, Drake DR 2011 Are introduced rats (*Rattus rattus*) both seed predators and dispersers in Hawaii? Biol. Invasions 13, 883–894. (10.1007/s10530-010-9876-7)

[108] Ware C, Bergstrom DM, Muller E, Alsos IG 2012 Humans introduce viable seeds to the Arctic on footwear. Biol. Invasions 14, 567–577. (10.1007/s10530-011-0098-4)

[109] Reim E, Blesinger S, Forster L, Fischer K 2018 Successful despite poor flight performance: range expansion is associated with enhanced exploratory behaviour and fast development*.* J. Evol. Biol. 31, 1165–1179. (10.5061/dryad.kg025m9) 29845691

[110] Tamburello N, Ma BO, Côté IM 2019 From individual movement behaviour to landscape-scale invasion dynamics and management: a case study of lionfish metapopulations. Phil. Trans. R. Soc. B 374, 20180057 (10.1098/rstb.2018.0057) 31352886 PMC6710579

[111] Silk MJ, Hodgson DJ, Rozins C, Croft DP, Delahay RJ, Boots M, McDonald RA. 2019 Integrating social behaviour, demography and disease dynamics in network models: applications to disease management in declining wildlife populations. Phil. Trans. R. Soc. B 374, 20180211 (10.1098/rstb.2018.0211) 31352885 PMC6710568

[112] Bailey DW, Gross JE, Laca EA, Rittenhouse LR, Coughenour MB, Swift DM, Sims PL 1996 Mechanisms that result in large herbivore grazing distribution patterns. J. Range Manage. 49, 386–400. (10.2307/4002919)

[113] Romare P, Hansson L-A 2003 A behavioral cascade: top-predator induced behavioral shifts in planktivorous fish and zooplankton. Limnol. Oceanogr. 48, 1956–1964. (10.4319/lo.2003.48.5.1956)

[114] Abrams PA 1984 Foraging time optimization and interactions in food webs. Am. Nat. 124, 80–96. (10.1086/284253)

[115] Beckerman AP, Uriarte M, Schmitz OJ 1997 Experimental evidence for a behavior-mediated trophic cascade in a terrestrial food chain. Proc. Natl Acad. Sci. USA 94, 10 735–10 738. (10.1073/pnas.94.20.10735) PMC23467 11038581

[116] Oro D, Genovart M, Tavecchia G, Fowler MS, Martinez-Abrain A 2013 Ecological and evolutionary implications of food subsidies from humans. Ecol. Lett. 16, 1501–1514. (10.1111/ele.12187) 24134225

[117] Kolowski JM, Holekamp KE 2008 Effects of an open refuse pit on space use patterns of spotted hyenas. Afr. J. Ecol. 46, 341–349. 10.1111/j.1365-2028.2007.00846.x

[118] Gilbert NI, Correia RA, Silva JP, Pacheco C, Catry I, Atkinson PW, Gill JA, Franco AMA 2016 Are white storks addicted to junk food? Impacts of landfill use on the movement and behaviour of resident white storks (*Ciconia ciconia*) from a partially migratory population. Mov. Ecol. 4, 7 (10.1186/s40462-016-0070-0) 26981250 PMC4791752

[119] Barton BT, Hodge ME, Speights CJ, Autrey AM, Lashley MA, Klink VP 2018 Testing the AC/DC hypothesis: rock and roll is noise pollution and weakens a trophic cascade. Ecol. Evol. 8, 7649–7656. (10.1002/ece3.4273) 30151178 PMC6106185

[120] MacGregor CJ, Evans DM, Fox R, Pocock MJO 2017 The dark side of street lighting: impacts on moths and evidence for the disruption of nocturnal pollen transport. Glob. Chang. Biol. 23, 697–707. (10.1111/gcb.13371) 27251575

